# Dyspnea in a Patient with Melanoma

**DOI:** 10.5811/cpcem.2018.11.41064

**Published:** 2019-01-07

**Authors:** Laura J. Bontempo, Nubia Seyoum

**Affiliations:** * University of Maryland School of Medicine, Department of Emergency Medicine, Baltimore, Maryland; † University of Maryland Medical Center, Department of Emergency Medicine, Baltimore, Maryland

## CASE PRESENTATION

**Image 1:**
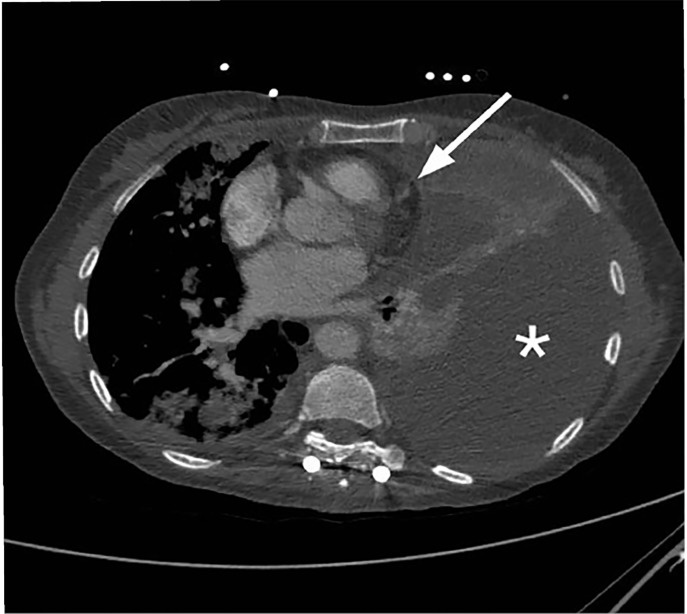
Thoracic computed tomography angiography axial view with large left pleural effusion (*) and rightward displacement of the heart (white arrow).

**Image 2:**
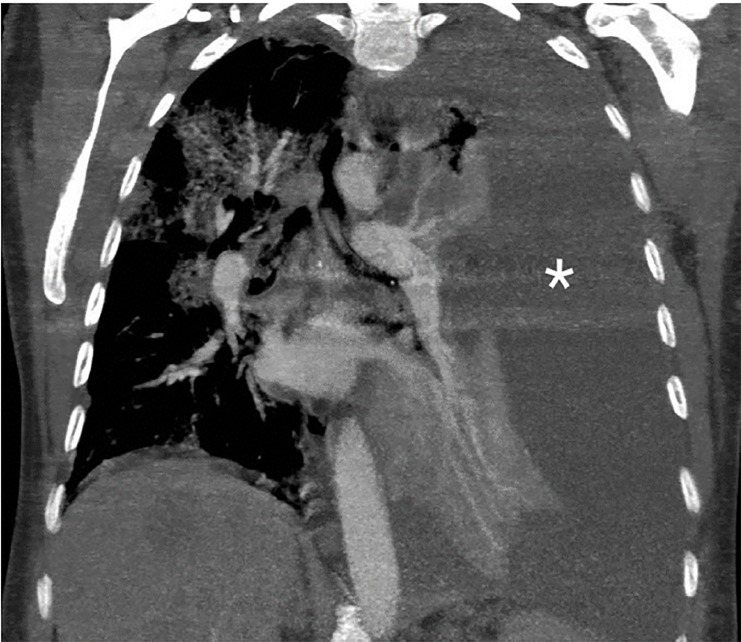
Thoracic computed tomography angiography coronal view with complete left hemithorax pleural effusion (*).

**Image 3:**
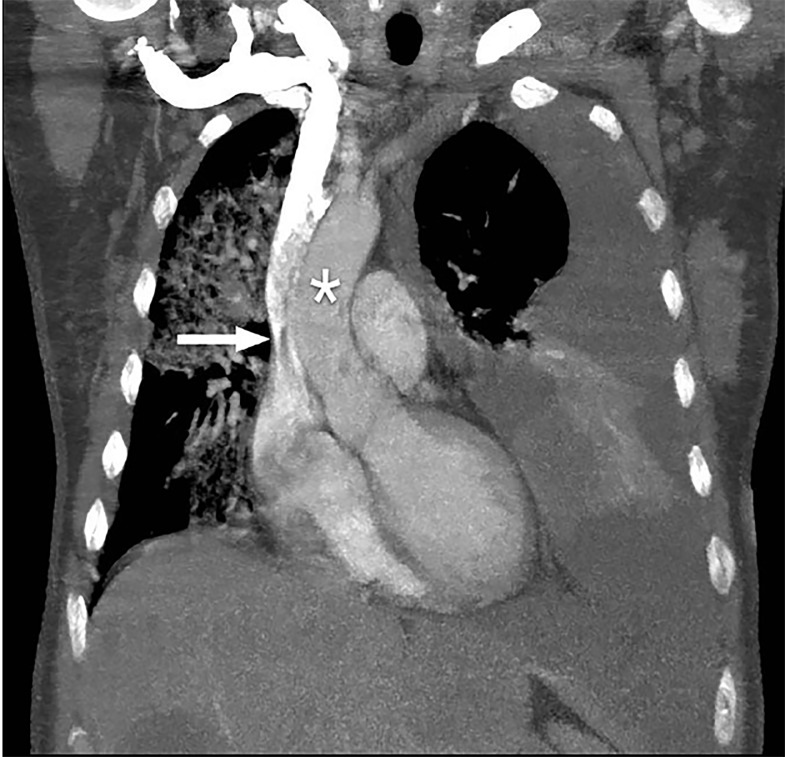
Thoracic computed tomography angiography coronal view with rightward mediastinal shift (*) and compression of superior vena cava (white arrow).

A 50-year-old woman with widely metastatic melanoma presented to the emergency department with dyspnea. She was found to be tachypneic, hypoxic, tachycardic, and hypotensive. A non-rebreather oxygen mask was placed and her oxygen saturation improved mildly. We obtained a semi-erect chest radiograph (CXR) followed by chest computed tomography angiography (CTA) (Images 1–3), due to concerns for a pulmonary embolism. The CXR revealed a depressed left hemidiaphragm and a left pleural effusion. The CTA revealed a massive left pleural effusion causing left lung atelectasis, rightward mediastinal shift, and depression of the left hemidiaphragm.

## DISCUSSION

The diagnosis was a tension hydrothorax due to a massive, malignant pleural effusion causing hemodynamic compromise. The hemodynamic effects of a tension hydrothorax are analogous to those of a tension pneumothorax. Elevated intrathoracic pressure caused by the effusion impairs venous return and compresses the left ventricle, causing reduced stroke volume and subsequent hypotension.^1^–^3^ Although pleural effusions are common, occurring in more than half of all cancer patients, a tension hydrothorax due to a massive pleural effusion is a rare event.^4^,^5^ It has been reported in cancer patients and as an iatrogenic complication of surgery.^1^,^4^–^6^

A CXR will show a large pleural effusion.^2^–^6^,^8^ However, as in our patient and in a previous case report,^6^ the CXR might indicate no, or only subtle, findings of intrathoracic tension. Tension findings are revealed by CTA. Emergent intervention is necessary to reduce intrathoracic pressure and allow venous return.^4^,^6^,^8^ In the management of our patient, we performed a left thoracentesis following intubation, allowing drainage of more than one liter of serosanguineous fluid. Her hemodynamics immediately improved. The patient eventually required thoracostomy tube placement for persistent hypoxia and risk of recurrent tension physiology.

The emergency physician must be aware of this entity when a hemodynamically-compromised patient arrives with a pleural effusion, even if mediastinal shift is not evident on the CXR.

**CPC-EM Capsule**

What do we already know about this clinical entity?

*A tension hydrothorax due to a massive pleural effusion is a rare event that can cause hemodynamic instability due to compromised thoracic venous return*.

What is the major impact of the image(s)?

*The images demonstrate how increased intrathoracic pressure can cause mediastinal shift and vena cava compression*.

How might this improve emergency medicine practice?

*In a hemodynamically compromised patient with a large pleural effusion, emergent thoracentesis should be considered due to possible tension physiology*.

## References

[1] Gouze VA, Breslow MJ (1996). Hydrothorax as a complication of percutaneous access to the renal pelvis. Anesth Analg.

[2] Chattranukulchai P, Satitthummanid S, Puwanant S (2013). A rare cause of pulsus paradoxus: acute tension hydrothorax. BMJ Case Rep.

[3] Deal A, Evans D, Counselman FL (2016). Tension hydrothorax from disseminated endometriosis. West J Emerg Med.

[4] Negus RA, Chachkes JS, Wrenn K (1990). Tension hydrothorax and shock in a patient with a malignant pleural effusion. Am J Emerg Med.

[5] Dixit R, Agarwal KC, Gokhroo A (2017). Diagnosis and management options in malignant pleural effusions. Lung India.

[6] Dagrosa RL, Martin JF, Bebarta VS (2009). Tension hydrothorax. J Emerg Med.

[7] Surani SR, Mendez Y, Anjum H (2016). Pulmonary complications of hepatic diseases. World J Gastroenterol.

[8] Azim A, Sahoo JN, Baronia AK (2012). Severe dengue with massive pleural effusion requiring urgent intercostal chest tube drainage: a case report. Am J Emerg Med.

